# Role of m^6^A in Embryonic Stem Cell Differentiation and in Gametogenesis

**DOI:** 10.3390/epigenomes4010005

**Published:** 2020-03-14

**Authors:** Lior Lasman, Jacob H Hanna, Noa Novershtern

**Affiliations:** The Department of Molecular Genetics, Weizmann Institute of Science, Rehovot 7610001, Israel

**Keywords:** RNA modification, *N*^6^-methyladenosine, m^6^A, stem cells, spermatogenesis, oogenesis

## Abstract

The rising field of RNA modifications is stimulating massive research nowadays. m^6^A, the most abundant mRNA modification is highly conserved during evolution. Through the last decade, the essential components of this dynamic mRNA modification machinery were found and classified into writer, eraser and reader proteins. m^6^A modification is now known to take part in diverse biological processes such as embryonic development, cell circadian rhythms and cancer stem cell proliferation. In addition, there is already firm evidence for the importance of m^6^A modification in stem cell differentiation and gametogenesis, both in males and females. This review attempts to summarize the important results of recent years studying the mechanism underlying stem cell differentiation and gametogenesis processes.

## 1. Introduction

Epigenetics, most commonly, refers to heritable changes in gene expression which are not linked to changes in the DNA sequence. As such, chemical modifications on DNA and histones were studied for decades, revealing the importance of epigenetic modifications in gene expression regulation. In recent years another level of gene expression regulation is under extensive research: the field of post-transcriptional RNA modifications, also known as the epitranscriptome [1]. These RNA modifications, which can be added post-transcriptionally, are another layer of gene expression regulation. For now, more than 170 modifications were found to be added post-transcriptionally to RNA molecules, the vast majority are on tRNA and rRNA [2]. *N*^6^-methyladenosine (m^6^A) is the most abundant modification on polyadenylated RNA [3] (Table 1).

**Figure 1:**
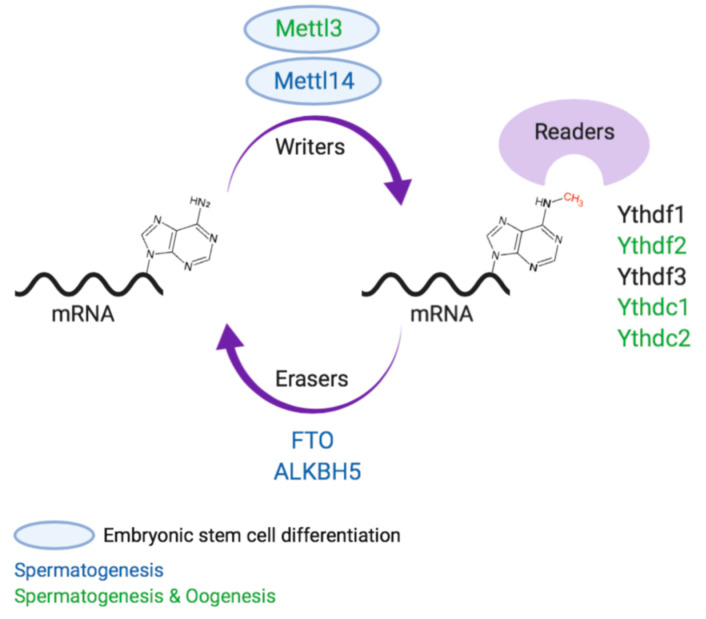
Conventions of m^6^A modification on mRNA, its writer, reader and eraser proteins. Proteins with evidence to have a role in embryonic stem cell differentiation, or in spermatogenesis or oogenesis, are marked in different colors.

RNA modifications were detected for the first time in the fifties of the last century [4,5]. However, only in the seventies, the most abundant mRNA modification was discovered—the *N*^6^-methyladenosine, also known as m^6^A, in poly(A) RNA fractions [6,7,8,9,10,11]. This modification, which consists of a methyl group added to adenosine (Figure 1) was found in prokaryotes, viruses, plants and mammals [12,13,14,15]; however, at that time there were no available methods to detect m^6^A sites on mRNA, thus scientist could not determine which specific RNA contains m^6^A modification. Hence, the research about the RNA modifications was delayed for a few decades.

In 2012, a leap forward was made in the research of m^6^A, in the form of the first-ever mapping of the sites of m^6^A modification in mammals, using a novel method of RNA-IP followed by high-throughput sequencing (MeRIP-Seq/m^6^A-seq) [16,17]. This method helped to stress the importance of m^6^A by demonstrating the conservation of its methylated sites during evolution [16,17,18,19]. In addition, by using this method, it has been shown that m^6^A modification occurs in specific sequence motif DRACH (D = A/G/U, R = G/A, H = A/C/U), and is preferentially localized around stop codons, 3′ UTRs regions and long exons [16,17,20]. Furthermore, these studies showed that the levels of m^6^A may be dynamic in certain settings, changing due to cellular stress and stages of development.

The notion that the m^6^A is a dynamic modification was further supported by the discovery of m^6^A demethylase FTO [21]. It was the first-ever confirmed reversible RNA modification, thus raising the attention to the field. Although nowadays, the latter result is controversial and it is also known that FTO is a demethylase of another modification—*N*^6^,2′-*O*-dimethyladenosine (m^6^Am) [22]—the idea that m^6^A modification is dynamically regulated is intriguing. Still, despite the great effort, there is no clear answer so far to whether m^6^A is dynamically regulated. m^6^A-seq and meRIP-seq methods are based on antibodies, and as such are limited in their ability to quantify methylation levels, and suffer from false positive hits. A recent work [23] presents a cleaner method for m^6^A mapping in a single-nucleotide resolution, and shows that the 33%–46% of the methylation is determined by the sequence alone, thus suggesting that the modification is less dynamic than suggested.

Regardless of this question, in recent years new discoveries demonstrated that mRNA modifications play a role in various molecular processes, such as translation efficiency, stability, localization and splicing, thus impacting cell fate [24,25,26,27,28,29,30]. Moreover, these modifications were shown to be crucial for biological processes such as embryonic development, cell circadian rhythms and cancer stem cell proliferation [19,31,32,33,34]. In this review, we focus on m^6^A mRNA modification and its role in mammalian stem and germ cells. For a review on m^6^A modification and its mechanisms, we refer you to other comprehensive reviews [35,36,37].

## 2. m^6^A Proteins

### 2.1. m^6^A “Writers”

In recent years the components necessary for writing m^6^A modification on RNA molecules were found (Figure 1), allowing the basic research of m^6^A modification. First to be found, and even before the development of the new methods, was the methyltransferase like 3 protein (METTL3), one part of a larger catalyzing complex [38,39]. METTL3 is widely expressed in different tissues in mouse and human, and when knocking-out METTL3 in mouse embryonic stem cells, no traces of m^6^A methylation can be found using mass spectrometry [31]. METTL3 was shown to play a major role in development and function of neural cells [40], cardiac cells [41,42], bone and muscle [43], hematopoiesis [44] and gametogenesis [45,46], as well as in cancer progression [47]. It was also shown in 2015 that it is essential for differentiation in vitro and for mouse development in vivo [31].

Later works discovered Wilm’s tumor-associated protein (WTAP) as an METTL3 adaptor protein, a crucial component of the m^6^A methyltransferase complex [48,49]. WTAP is important for the complex function and when knocked-down, m^6^A levels in human cells are decreased significantly [25].

Another protein was suggested to be an m^6^A writer—METTL14 protein. It was shown that METTL3 and METTL14 work in a complex [20]. As such, knock-down of METTL14 also leads to decreased m^6^A levels [20,25]. Knock-out (KO) of METTL3 or METTL14 results in protein loss of the other writer [31,50], suggesting a coupled stoichiometry. Recent papers demonstrate that METTL14 is not enzymatically active, thus does not have a role as a second methyltransferase, but as an adaptor, helping METTL3 by facilitating RNA binding [51,52,53].

With time, several more proteins have been shown to be important for facilitating the function of the methyltransferase complex and its correct positioning. This includes RNA binding motif protein 15/15B (RBM15/15B), which binds the methylation complex and was shown to methylate XIST [54], Vir like m^6^A methyltransferase associated (VIRMA), which recruits the methylation complex to 3′ UTR [55], and zinc finger CCCH-type containing 13 (ZC3H13), which modulates the activity of the complex in the nucleus [56]. In addition, METTL16 was discovered as another m^6^A methyltransferase which adds m^6^A in a different sequence context and is important for splicing [57,58].

### 2.2. m^6^A “Erasers”

There are two proteins which were found to function as m^6^A mRNA demethylase enzymes (Figure 1)—FTO [21] and α-ketoglutarate-dependent dioxygenase alkB homolog 5 (ALKBH5) [59]. These two m^6^A erasers have been found to reduce m^6^A levels following over-expression, and increase m^6^A levels by 9%–23% following knock-down. The presence of these m^6^A erasers suggests that m^6^A modifications can be dynamic and reversible, at least to some extent, in a similar manner to DNA and protein modifications.

However, as mentioned before, the role of FTO as an m^6^A demethylase is still under debate. It was found that FTO preferentially demethylases m^6^Am modification, rather than the m^6^A modification [22]. In 2018 it was shown that FTO bind multiple RNA species and function as demethylase of m^1^A, m^6^A and m^6^Am [60]. In addition, little is known about ALKBH5. Interestingly, ALKBH5-KO mice are viable and normal except for defects in spermatogenesis [59].

### 2.3. m^6^A “Readers”

The actual effect of m^6^A modification is mediated by “readers”, that are m^6^A binding proteins (Figure 1). m^6^A reader proteins which predominantly bind to methylated RNA were initially identified in RNA pull-down experiments, including YTHDF2 and YTHDF3 [16]. These cytoplasmatic proteins belong to the YT521-B homology (YTH) domain. Since then, more groups have shown using crystallography and gel shift assays that YTH proteins, including YTHDF2 and YTHDC1, in addition to yeast homolog MRBP1, are m^6^A binding proteins [61,62,63], eventually concluding that all cytoplasmic YTH domain family (YTHDF1, YTHDF2 and YTHDF3), and nuclear YTH domain containing proteins (YTHDC1, YTHDC2), function as m^6^A binding proteins [64,65]. YTH domain interacts with methylated adenosine with low affinity, using an aromatic cage that surrounds the methyl group, as concluded from structure studies of MRBP1, YTHDC1 and YTHDF1/2 [64]. Unfortunately, the crystal structure of YTHDF3 has not been achieved so far.

Studies on YTHDF2 demonstrated that its binding motif is identical to the m^6^A motif, and that by binding to this motif, YTHDF2 regulates m^6^A-dependent RNA degradation through interaction with CCR4-NOT deadenylase complex [66,67]. YTHDF1 and YTHDF3 were suggested to promote mRNA translation through recruiting translation initiation factors [68,69]. YTHDC1 was linked to mRNA splicing, export and degradation [70,71,72]. YTHDC2 mediates translation and decay specifically in spermatogenesis [65].

With time, more m^6^A readers were discovered, including HNRNPC/G, HNRNPA2B1, IGF2BP1-3 and FMR1. These proteins contain other types of RNA-binding domains, and a different recognition site (e.g., IGF2BP1-3 bind to GG(m^6^A)C). They were suggested to be important for mRNA splicing, stability and translation [73,74,75,76,77,78,79]. In 2015 it was suggested that the binding mechanism of HNRNPC/G is different than that of YTH containing readers: the methylation induces a structural change to the mRNA, thus exposing the binding site of HNRNPC/G/A2B1 and facilitating their binding [74]. Interestingly, some m^6^A binding proteins may work in a competitive manner. For instance, YTHDF2 and IGF2BP1-3 affect mRNA stability in an opposite manner [76]. In this case, they also have different binding preferences—YTHDF2 tends to bind 3′ UTR, and IGF2BP1-3 tends to bind CDS [76]. It is therefore interesting to understand the mechanism that orchestrate the activity of these readers.

m^6^A modification plays a major role in differentiation. It is therefore natural to use stem cell differentiation, a model which is easy to manipulate, to investigate the different aspects of m^6^A. Here we will summarize the work that was done to investigate m^6^A in the context of embryonic stem cells.

## 3. m^6^A and Stem Cell Fate

Embryonic stem cells are a unique type of cells, which reside in the early embryo blastocyst, and give rise to all the tissues of the embryo (“pluripotent”). These cells can self-renew, and can be maintained in vitro indefinitely in their pluripotent state. Embryonic stem cells are typically divided into two states—“naïve” state, which is found in the blastocyst inner cell mass, and “primed” state, which is primed to differentiation, and is found in the epiblast. Naïve and primed cells are distinguished in several molecular parameters such as inactivation of X chromosome, activity of OCT4 enhancers and the signaling required for their in vitro maintenance [80]. Key transcription factors such as NANOG, OCT4, SOX2, ESRRB, KLF4, TFAP2C and others form an interconnected circuit orchestrating pluripotency [81].

In 2014, Batista and colleges showed that core pluripotency transcription factors are methylated in mouse and human [19]. Depletion of mouse and human METTL3 was shown to reduce significantly the m^6^A deposition, resulting in prolonged NANOG and SOX2 expression upon differentiation, and impaired exit from pluripotency [19,31]. By studying m^6^A modification in vivo using METTL3-KO mice, our group has demonstrated that depletion of m^6^A in mice is embryonic lethal [31]. The embryos die between embryonic day 3.5 and 6.5, the exact stage in which the cell in the blastocyst inner cell mass are exiting from pluripotent state and starting to differentiate. Teratoma and EB assays confirmed that cells that lack m^6^A are in a “hyper-pluripotent” state, and had a poor differentiation ability [31]. The erasure of m^6^A from some of the pluripotent transcripts such as NANOG, SOX2, ZFP42, KLF4 and others, resulted in prolonged half-life. As a consequence, pluripotent circuitry was not turned off, thus emphasizing the importance of m^6^A in the exit from naïve pluripotent state [31]. Notably, knock-out of METTL14 in mouse ESCs recapitulates METTL3-KO phenotype in vivo and in vitro [82].

Other groups reported that mouse embryonic stem cells with knockdown of METTL3 and METTL14 have lost their self-renewal capability and overexpress developmental regulators [24]. This seeming contradiction can be resolved by the notion that lack of m^6^A affects the genes that are currently expressed. In the naïve conditions [80], the cells will stay pluripotent, and in primed conditions, in which the cells are primed to differentiation, the cells will lose their self-renewal and differentiate.

Overall, when large-scale changes in the cell are required, such as in differentiation or programming, m^6^A has an important role in the proper regulation of mRNA. This has been shown in cellular reprogramming, which m^6^A promotes [83], and during differentiation. For instance, lincRNA1281 was shown to have a crucial role in mESCs differentiation by sequestering let-7 miRNA [84], and this interaction is mediated by m^6^A modification. In 2018 a novel mechanism of m^6^A deposition regulation was uncovered by using human embryonic stem cells model. By investigating the interactome of SMAD2/3 during differentiation of human primed pluripotent stem cells, it was shown that SMAD2/3 interacts with the METTL3-METTL14-WTAP complex [85]. Nonetheless, by using nuclear-enriched m^6^A-methylated-RNA immunoprecipitation followed by deep sequencing, it was demonstrated that activin/TGFβ signaling, mediated by SMAD2/3, promotes m^6^A deposition on nuclear RNAs. The upregulation of m^6^A deposition occurs on several transcripts, including transcripts such as NANOG, NODAL and LEFTY1, which are activin signaling targets and function as pluripotency regulators. Thus, inhibition of activin/TGFβ signaling leads to differentiation, meaning that regulation of m^6^A deposition can be dynamically changed by extracellular signaling [85].

m^6^A is important for the differentiation of adult stem cells as well. During neural stem cell differentiation, YTHDF2 is responsible for adequate decay of mRNAs that are inhibitory of Jak/Stat cascade [86]. Upon knock-out of YTHDF2, NPCs are unable to produce functional neurites. Other mechanisms that are important for neural differentiation are dependent on m^6^A [87,88]. Hematopoiesis is also dependent on m^6^A modification. Hematopoietic stem cells (HSCs) that lack m^6^A methylation (METTL3-KO) are unable to differentiate, and are accumulated in the bone marrow [44]. Transcriptional analysis revealed that knock-out cells are unable to upregulate m^6^A-methylated MYC expression, where enforced upregulation of MYC rescued the phenotype [44]. YTHDF2-KO promotes HSCs expansion as well, what has important medical implication [89]. In 2019 it was shown that MYC is responsible for symmetric committed differentiation of HSCs and that upon METTL3-KO, the reduction of MYC brings the cells to be blocked in a multipotent progenitor-like state [90]. 

To conclude, regulation of mRNA by m^6^A modification is essential for proper differentiation of embryonic and adult stem cells by multiple pathways. For further reading on m^6^A in pluripotency and development we refer the readers to the review by Heck and colleges [91].

## 4. Gametogenesis

Gametogenesis, the process of gametes generation, is another differentiation process which is heavily mediated by m^6^A modification. Primordial germ cells are the cells that give rise to the gametes of an organism that reproduces sexually. During gametogenesis, these cells undergo meiosis, followed by cellular differentiation into mature gametes—oocytes in females or spermatozoa in males. Early works showed that m^6^A is essential for proper meiosis in yeast [18,92,93]. It is therefore intriguing to test gametogenesis, the process in which meiosis happens in mammals, and is accompanied by massive degradation of RNA.

### 4.1. m^6^A Role in Spermatogenesis

**Figure 2:**
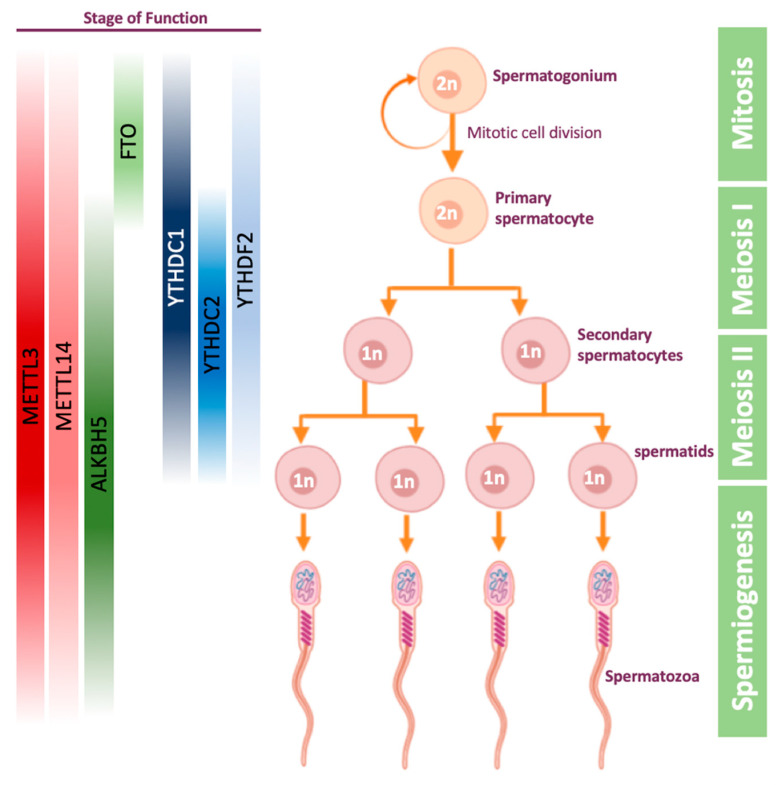
Stages of spermatogenesis, and the proteins that have a role during the process. The proteins are highlighted along the stages in which there is some evidence that they play a role. Other stages are yet to be confirmed.

Spermatogenesis is a process undergoing in the seminiferous tubules of the testis, in which germ cells develop into haploid spermatozoa. This process starts with the mitotic division of spermatogonial stem cells, which can either self-review, or give rise to primary spermatocytes (Figure 2). The latter cells then undergo the first meiosis to become secondary spermatocytes. The secondary spermatocytes undergo the second meiosis, and by the end of this process they become haploid spermatids. In the final stage of spermatogenesis, called spermiogenesis, spermatids are transformed into mature and motile spermatozoa, also known as sperm cells (Figure 2).

The first evidence for the importance of m^6^A on gametogenesis was demonstrated in male mice: erasing m^6^A modification by ALKBH5 was shown to impact mouse spermatogenesis [59]. It was demonstrated that ALKBH5 is highly expressed in mice testis, and that loss of ALKBH5 leads to differentially expressed genes related to spermatogenesis [59]. ALKBH5-deficient male mice were shown to have increased m^6^A levels on mRNA, significantly smaller testes compared to their WT littermates and impaired fertility due to apoptosis which defects the meiotic metaphase-stage spermatocytes [59]. In 2017, ALKBH5-dependent m^6^A demethylation was shown to be critical for splicing and stability of 3’ UTR mRNAs in spermatocytes and round spermatids [94]. Later, it was demonstrated that inhibition of FTO using meclofenamic acid leads to an increased level of m^6^A and decreased expression of few cyclin-dependent kinases, resulting in abnormal spermatogonial proliferation [95].

The understanding that m^6^A plays a role in spermatogenesis led to other works on writer and reader proteins. It was demonstrated that METTL3 and METTL14 are localized in the nuclei of germ cells in mice testis [96]. Conditional mice knock-out of METTL3 or METTL14 using VASA-Cre line, which activates the knock-out early in migratory primordial germ cells [97], were sterile, with significant reduction of m^6^A levels in undifferentiated spermatogonia. This led to spermatogonial stem cells defects, causing translational dysregulation of methylated transcripts, including genes that are crucial for proliferation and differentiation of spermatogonial stem cells [96], overall showing that depletion of a single methyltransferase is sufficient to cause a sever phenotype in male mice. In another study, single methyltransferase knock-out mice, METTL3^f/f^VASA-cre+, were found to have abnormal meiosis initiation and spermatogonial differentiation [46]. These mice were normal in size but completely infertile, with 80% reduction in testis weight at eight weeks old. Furthermore, defects in sperm maturation and motility upon conditional METTL3-KO were detected also in zebrafish, establishing that m^6^A modification is essential for spermatogenesis outside the mammalian domain [45].

Out of all reader proteins, the protein which studied the most in the context of spermatogenesis, is YTHDC2. YTHDC2 was found to be essential for mouse meiosis, both in males and females, in several different papers [65,98,99,100]. YTHDC2-KO male mice have significantly smaller testes and female mice have significantly smaller ovaries, compared to their littermates [65]. However, it is still unclear whether these mice are sterile due to YTHDC2 ability to recognize the m^6^A modification using its YTH domain or due to its 3’→5’ RNA helicase activity. YTHDC2 was shown to be expressed in the cytoplasm of mice spermatocytes and interacts with MEIOC, which is an important gene in the meiosis process [101]. When YTHDC2 is knocked-out in male mice, the meiosis is initiated properly but continues aberrantly, finally leading to apoptosis. In addition, it was demonstrated in transcriptome analysis that when YTHDC2 is missing, genes associated with meiosis are downregulated and genes associated with mitotic cells are upregulated, indicating YTHDC2 is essential for ending mitosis and transition to meiotic process [98,99,100].

YTHDC1 is essential for proper embryonic development—YTHDC1-KO mouse embryos die at early post-implantation stages [102]. YTHDC1 is expressed in the nucleus of spermatogonia, spermatocytes, and round spermatids, which are transcriptionally active during spermatogenesis [102]. In addition, YTHDC1^f/−^ VASA-Cre+ mice have reduced spermatogonia on day 8 (post-natal), and no germ cells in their testis from day 25 post-natal and on, indicating that YTHDC1 is not important only for mouse viability, but also for the spermatogenesis process [102].

Few observations yet connect YTHDF readers to spermatogenesis. A work from 2017 showed that YTHDF2-KO male mice are fertile, in contrast to YTHDF2-KO females, and have normal seminiferous tubule histology [103]. Depletion of YTHDF2 leads to downregulation of matrix metallopeptidase, thus affecting cell adhesion and proliferation. This phenotype can be partially rescued by inhibition of MMP13 [104]. However, further examination of all YTHDF readers in spermatogenesis is still required.

### 4.2. m^6^A Role in Oogenesis

**Figure 3:**
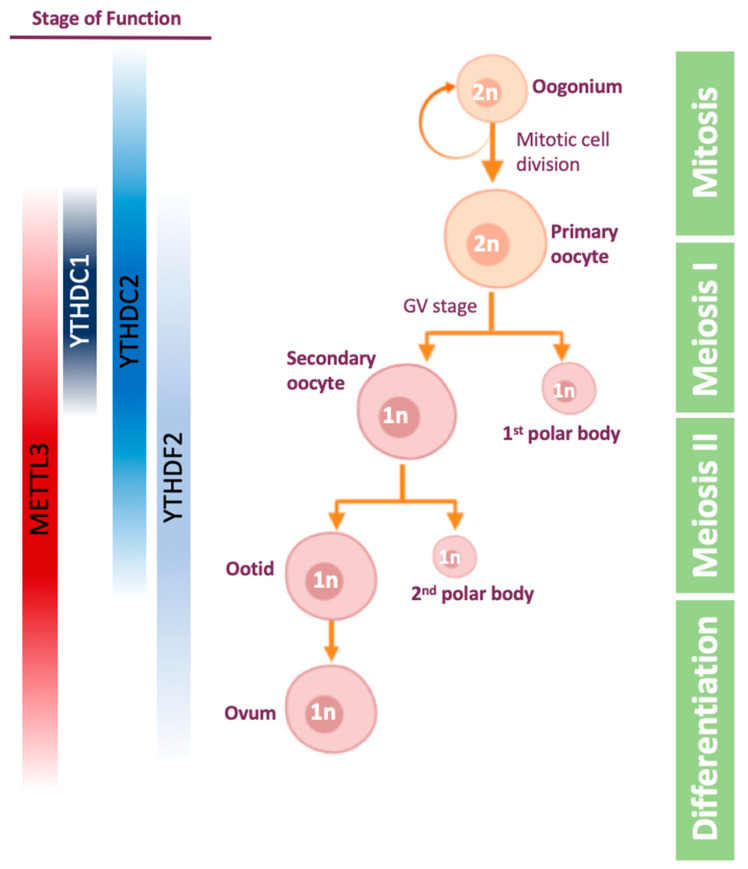
Stages of Oogenesis, and the proteins that have a role during the process. The proteins are highlighted along the stages in which there is some evidence that they play a role. Other stages are yet to be confirmed.

The oogenesis process (Figure 3) is the differentiation of the diploid germ cell, called oogonium, into an ovum (egg cell). The oogonium undergoes transformation into primary oocyte in a process called oocytogenesis. The primary oocyte enters the first meiosis and is arrested in prophase I in germinal vesicle (GV) stage. Following hormonal trigger, the oocyte resumes the first meiosis and becomes secondary oocyte. Then, the secondary oocyte undergoes the second meiosis division and is arrested in metaphase II. If the secondary oocyte is fertilized, meiosis II is resumed and completed.

The maternal mRNA is transcribed and accumulated during the oocytes’ growth, and transcription ends in GV stage oocytes [105]. Later on, mRNAs are massively degraded when the oocytes proceed the meiosis process following a hormonal trigger [106,107]. The mRNA degradation goes on after fertilization as well, and as a result most of the maternal mRNA is degraded in the 2-cell stage [108]. Thus, mRNA stability appears to be a crucial process in oogenesis and maternal to zygotic transition, highlighting the importance of post-transcription regulation during these processes.

The first evidence for m^6^A importance during these processes in females was observed in zebrafish, showing that one-third of maternal mRNA is m^6^A-methylated, and its degradation, mediated by YTHDF2 activity, is important for maternal to zygotic transition and timely development of the zebrafish [109]. A later paper on zebrafish showed that loss of METTL3 results in failed gamete maturation and reduced fertility [45]. In mammals as well, YTHDF2 reader protein was the first link found between m^6^A and oogenesis: YTHDF2 is expressed throughout mouse oogenesis, as demonstrated by immunostaining [103]. Knocking-out YTHDF2 leads to normal ovulation but an inability to downregulate maternal mRNA. As a result, approximately 270 genes are deregulated and mouse embryo development is defected at or prior to 2-cell stage, leading to infertility [103].

Studying the effect of m^6^A writers on oogenesis was more challenging due to the fact that METTL3-KO is embryonic lethal in mice [31]. While in spermatogenesis studying this difficulty was solved by using conditional knock-out Cre, METTL3-knock-down female mice were generated by microinjections of siRNAs or morpholino into GV oocytes [110]. The knock-down resulted in reduced translation efficiency, defects in mRNA degradation, and impaired meiotic maturation, including aberrant spindle organization. Overall, it was demonstrated that m^6^A is crucial for oocyte maturation and maternal-to-zygotic transition [110].

As mentioned before, YTHDC2 also affects oogenesis. It was shown that YTHDC2-KO mice are infertile as their oocytes cannot develop beyond the zygotene stage of meiotic prophase I [65,98,99]. Another m^6^A reader that was found to be important for mouse oocyte and spermatogonial development, is the nuclear factor YTHDC1. Loss of YTHDC1 leads to extensive polyadenylation and altered 3′ UTR length, in addition to defective alternative splicing [102]. As a result, there are no secondary or antral follicles in the ovaries, indicating YTHDC1-deficient oocytes are blocked at the primary follicle stage. Indeed, YTHDC1 interacts with pre-mRNA 3′ end processing factors CPSF6, SRSF3 and SRSF7 [102].

## 5. Discussion

To conclude, many discoveries about m^6^A and its function in stem cells and germ cells were made in recent years. It is clear that m^6^A play a crucial role when orchestrated major changes in transcription are required, such as in embryonic development, differentiation of adult stem cells and gametogenesis. Looking to the future, much more research is required in order to fully understand the mechanism underlying these processes.

Two main related questions have not been fully answered. First is to what extent the modification is dynamic—on one hand there are multiple proteins which write, read and erase the modification, which suggests the modification is dynamically regulated in different cellular contexts. On the other hand, so far there is no work proving that the modification stoichiometry is changing between cellular states. As mentioned above, one of the reasons for the lack of such work, is that m^6^A-seq and meRIP-seq, based on antibodies, are limited in their ability to quantify methylation levels. A recent work that is using a cleaner method for m^6^A mapping in single-nucleotide resolution [23], shows that the methylation is in large not dynamic. However, even in the scenario where modification is not dynamically regulated, it is clear that its role for proper differentiation in vitro and in vivo is indispensable. The second related question is how specificity is achieved during writing, reading and erasing m^6^A. It is likely that the different m^6^A proteins interact with other proteins which regulate their activity, and may even direct them to their targets, thus generating specificity. However, more research is required to find those mechanisms.

More specifically, the interplay between YTHDF1, YTHDF2 and YTHDF3 should be studied. The three proteins are highly similar, and share many of their targets [64]. Is there a redundancy between the function of the readers? How do these m^6^A readers achieve specificity of their targets during gametogenesis and during stem cell differentiation? Are they expressed in different stages or in different levels? Furthermore, a comprehensive understating of other m^6^A related proteins in oogenesis and spermatogenesis is still missing.

It is also intriguing to investigate the role of m^6^A in human gametogenesis models. Loss of function of YTHDF2 was not detected in human population (pLI score = 1), however, it is interesting to check whether some cases of human infertility can be associated with aberrant m^6^A regulation.

## Figures and Tables

**Table 1:** Known mRNA modifications.

Name	Short Name	Formula	Types of Modified RNA
*N*^6^-methyladenosine	m^6^A	C_11_O_4_N_5_H_15_	mRNA, rRNA, snRNA, tRNA
*N*^6^-formyladenosine	f^6^A	C_11_H_13_N_5_O_5_	mRNA
*N*^6^,2′-*O*-dimethyladenosine	m^6^Am	C_12_O_4_N_5_H_17_	mRNA, snRNA
*N*^6^-hydroxymethyladenosine	hm^6^A	C_11_H_15_N_5_O_5_	mRNA
5-Methylcytidine	m^5^C	C_10_O_5_N_3_H_15_	mRNA, rRNA, tRNA
5-Hydroxymethylcytidine	hm^5^C	C_10_O_6_N_3_H_15_	mRNA
7-Methylguanosine cap (cap 0)	m^7^Gpp(pN)	C_11_H_15_N_5_O_11_P_2_	mRNA, snRNA
Inosine	I	C_10_O_5_N_4_H_12_	mRNA, tRNA
Pseudouridine	ψ	C_9_O_6_N_2_H_12_	mRNA, mRNA, rRNA, snRNA, snoRNA, tRNA
*N*^1^-mthyladenosine	m^1^A	C_11_O_4_N_5_H_15_	mRNA, rRNA, tRNA
